# A Low-Power High-Accuracy Urban Waterlogging Depth Sensor Based on Millimeter-Wave FMCW Radar

**DOI:** 10.3390/s22031236

**Published:** 2022-02-06

**Authors:** Hanyue Shui, Haoran Geng, Qiong Li, Li Du, Yuan Du

**Affiliations:** School of Electronic Science and Engineering, Nanjing University, Nanjing 210001, China; 191180108@smail.nju.edu.cn (H.S.); hrgeng@smail.nju.edu.cn (H.G.); 181180059@smail.nju.edu.cn (Q.L.)

**Keywords:** urban waterlogging depth, mmWave FMCW radar, SFCC, a complete and practical system, edge computing

## Abstract

The method of making precise measurements of remote water depth using mmWave technology has great potential for preventing urban waterlogging. To achieve waterlogging prevention, the mmWave system needs to measure the water depth change accurately with a short acquisition time. This paper demonstrates a new accurate mmWave water depth measurement system based on Frequency Modulated Continuous Wave (FMCW) Radar with a center frequency of 77 GHz. To improve distance resolution and lower acquisition time, the Swept Frequency-Cross Correlation (SFCC) algorithm is proposed for the first time to improve the distance computation resolution by 9× and lower time complexity from O(n·logn) to O(n) compared to traditional FFT-based FMCW radar distance computation. A prototype system equipped with a humidity sensor, a processor module and TI’s FMCW radar module is designed for monitoring urban floods in cities. Using the prototype system with the proposed SFCC, the depth measurement error is reduced from 4.5 cm to less than 5 mm, compared to the default radar post-processing algorithm embedded in the radar module.

## 1. Introduction

For decades, urban waterlogging has been considered a serious problem for cities since it causes not only traffic interruption but also the loss of property and life [1]. To reduce the impact and potential effect of waterlogging, a complete waterlogging monitoring system is necessary, as it can provide real-time information about the water depth of the city, adding support for dispatch, rescue and reasonable travel guides.

The urban waterlogging monitoring system often comprises four parts, including a data acquisition system, data transmission equipment, a control center and a user terminal. Currently, multiple data acquisition systems are in use, with data sources coming from water-level sensors [2,3], remote sensing data [4,5,6], social-media data [7,8,9,10], and video surveillance data [11,12,13,14]. However, the foremost problem arising in these approaches is the fact that the measurements can be affected by the environment to varying extents. Water and silt may pollute and cause damage to water-level sensors, and temperature or noise can impact infrared or ultrasonic-type remote sensing data, while social-media/crowdsourcing data and video surveillance data will be easily influenced by rain, snow, fog and night-darkness [15]. To provide a robust measurement of the water depth in various environments, the mm-Wave FMCW radar is proposed as it is not affected by poor visibility or certain harsh environmental conditions, making it a proper method for waterlogging monitoring when compared with other methods mentioned above [16].

The measurement of the water depth comes from the fundamental concept of radar ranging, which is the transmission of an electromagnetic signal that objects reflect in its path so that two values of distance can be obtained with the two reflective surfaces of water and ground, respectively. The FMCW radar transmits chirps captured by the receiving antenna (RX ant) combine with the TX signal to produce the beat frequency $f_{b}$ or intermediate frequency (IF) in a frequency mixer. Then, IF is sampled to make raw IF data.

**Figure 1:**
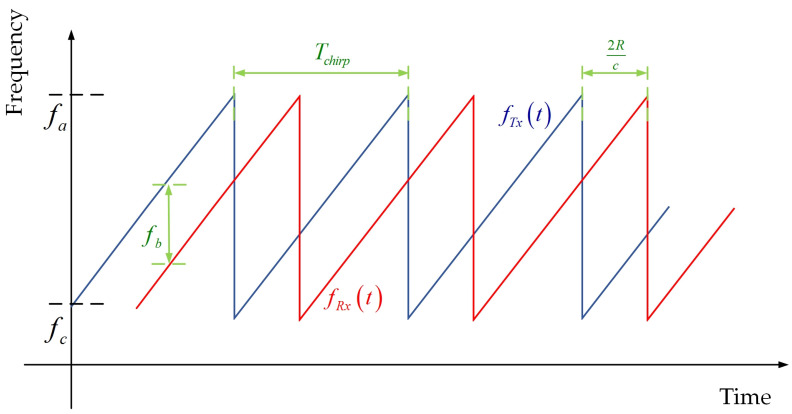
Normal pattern of mmWave FMCW radar.

With frequency as a function of time, the most often used mmWave FMCW signal pattern is shown in Figure 1.

The transmitted signal $f_{Tx}\left(t\right)$ whose frequency is swept linearly from a center frequency $f_{c}$ to a maximum frequency $f_{a}$ within a certain period ($T_{chirp}$) given by $S_{T}\left(t\right)$ is defined as:

(1) $$S_{T}\left(t\right)=\cos\left(2\pi f_{c}t+\pi \frac{BW}{T_{chirp}}t^{2}\right),$$

where BW = $\left(f_{a}-f_{c}\right)$ represents the bandwidth of a chirp.

Notice that the RX signal given by $S_{R}\left(t\right)$ is a time-delay version of the TX signal:

(2) $$S_{R}\left(t\right)=S_{T}\left(t-\frac{2R}{C}\right),$$

in which *R* is the distance to the measured object and c is the speed of light. Therefore, the IF signal is a cosine wave given by $S_{IF}\left(t\right)$ defined as:

(3) $$S_{IF}\left(t\right)=\cos\left(2\pi \frac{BW\times 2R}{T_{chirp}\cdot c}t+2\pi f_{c}\frac{2R}{c}\right),$$

of which the frequency and the phase are both proportional to the round-trip delay and are used for distance estimation after the ADC block, typically by using FFT. The distance resolution $d_{Res}$ is decided by BW:

(4) $$d_{Res}=\frac{c}{2BW},$$

For a regular FMCW radar module such as Texas Instruments (TI) IWR1443Boost, the maximum BW is around 4 GHz, resulting a $d_{Res}$ of 3.75 cm, which is not enough for water depth measurements. In addition, edge computing for FMCW-based depth computation is required as the waterlogging in cities caused by heavy rain is usually combined with a bad wireless communication signal, which limits the potential of sending data to the cloud for cloud computing [17,18,19]. The radar receives 8,388,608 data for measuring at once. Without IoT, all data the radar receives need to be sent to the cloud, while only the result of the depth needs to be sent to the cloud if IoT is used to process data and calculate the depth. In order to provide accurate depth measurement with relevant short processing time, new local radar processing algorithms are required to achieve low computation time complexity and power consumption, while generating depth measurement accuracy simultaneously.

Several studies on the algorithms of FMCW mmWave radar systems have been proposed [20,21,22,23,24,25,26,27,28,29,30]. Most of the proposed algorithms are based on Fast Fourier Transform (FFT), but it is difficult for FFT-based estimators to distinguish between two adjacent targets, such as ground and the water surface, because of the limitation of the bin width of the FFT spectrum given by $F_{s}/N$, in which $F_{s}$ and N, respectively, denote the sampling frequency and the number of samples [20]. One way to increase frequency resolution is zero padding used to interpolate the FFT spectrum. However, zero padding not only leads to an increase in complexity, but also has limitations when two targets’ corresponding peaks are within the 3-dB width of the window function. In [21], Adaptive Mirror Padding and Phase Correction Padding related to zero padding are proposed to improve $\rho_{s}$, but the error is around 10 cm, which can not satisfy the requirement of measuring water depth. The research in [22] studies a distance estimation scheme exploiting the deep learning technology of artificial neural networks to replace FFT spectrum analysis to obtain more accurate distance measurement. However, it also adds more computation complexity and can only achieve a resolution of 6.9 cm, which is insufficient for waterlogging monitoring. In [20] combining frequency and phase estimation, the resolution is around a few microns, but the applicable distance range is 5 mm, which is too small for our system. In [23], a novel direction of arrival (DOA) estimation algorithm is proposed employing the FFT and achieves very low computational complexity. Although its resolution is higher than that of conventional FFT-based estimation algorithms, it is still a few centimeters without much improvement [24]. Other algorithms [25,26,27,28,29,30], such as the multiple signal classification (MUSIC) [27], and estimation of signal parameters via rotational invariance technique (ESPRIT) algorithms [29] are also proposed. However, those super-resolution algorithms all add too much computing complexity, which is opposed to the conception of edge computing.

For the waterlogging monitor system, with the features of the application scenarios being short-distance and high-resolution detection, we propose an algorithm called SFCC to overcome the above limitation, increase the detection resolution of the FMCW radar and reduce computing time. In addition, a complete and practical system equipped with a humidity sensor, a processor module, and a display module is built based on edge computing to demonstrate the possibility of urban waterlogging monitors using IoT devices; The main contributions of this paper include:

- A new algorithm based on cross correlation, called SFCC, is invented where the frequency and phase information are combined to achieve high distance detection resolution, which is less than 5 mm with time complexity being O(n), while that is O(n·logn) for FFT and $O\left(n^{2}\right)$ for cross correlation;
- A prototype low-cost system with edge computing embedded is designed to demonstrate the potential of using SFCC in the IoT device to automatically monitor urban floods;
- Event-driven operation is implemented in the prototype system so that standby mode and measuring mode can be switched automatically according to the environmental change, achieving more than 100× power consumption reduction compared to always-on mode operation.

## 2. SFCC Algorithm

To increase the detection resolution of the FMCW radar and reduce computing time, we invent SFCC algorithm, combining the frequency and phase information based on cross-correlation calculation.

### 2.1. SFCC Algorithm Introduction

The core of the SFCC algorithm is cross-correlation calculation. The result of cross-correlation $R_{fh}\left(x\right)$ between two functions f(x) and h(x) is defined by the infinite integral with parameter variable x:

(5) $$R_{fh}\left(x\right)=\int_{-\infty }^{\infty }f*\left(x^{\prime }\right)h\left(x^{\prime }+x\right)dx^{\prime },$$

or

(6) $$R_{fh}\left(x\right)=\int_{-\infty }^{\infty }f*\left(x^{\prime }-x\right)h\left(x^{\prime }\right)dx^{\prime },$$

Physically, the result of cross correlation reflects the similarity between two signals. In signal analysis, cross correlation represents the correlation degree of two different time series at any different time, denoting whether the two signals are related in the frequency domain.

**Figure 2:**
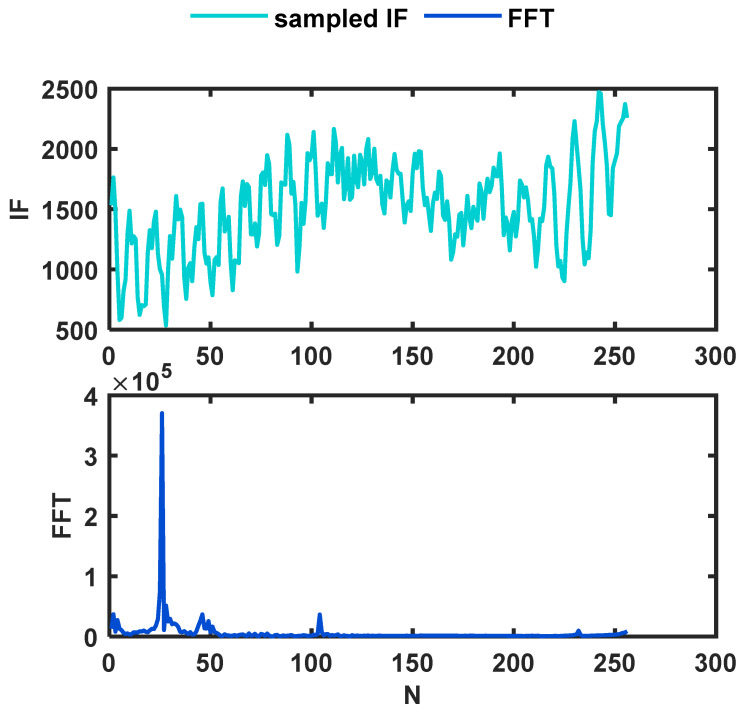
Sampled IF of time domain and frequency domain.

As shown in Figure 2, if various interference signals are ignored, the sampled signal is close to a single-tone signal. Therefore, cross correlation is used to find the value of the frequency closest to the sampled IF signal, which can convert the frequency domain analysis into time domain analysis and make fuller use of the information about the sampled signal than FFT.

### 2.2. Algorithm Initialization

In the specific application scenario, to achieve expected computing time, it is necessary to determine the frequency range of the single-tone signal ($F_{min}\;\mathrm{to}\;F_{max}$) to be calculated by cross-correlation with the sampled received signal and the phase range when finding the maximum correlation between two signals according to the approximate distance range.

#### 2.2.1. Frequency Range

After IF is sampled by ADC, $f_{IF}$ is given by the following equation:

(7) $$f_{IF}=F_{d}\times f_{s},$$

in which $F_{d}$ denotes the normalized digital frequency of IF and $f_{s}$ represents the sampling frequency. Therefore, the maximal value of the frequency $F_{max}$ can be calculated by the maximal distance from the radar module to the ground ($R_{gmax}$) combining Equations (3) and (7):

(8) $$F_{max}=\frac{BW\times 2R_{gmax}}{T_{chirp}\times c\times f_{s}\;}.$$

Furthermore, the range of the frequency of the single-tone signal $\Delta F_{d}\;$=$\;F_{max}-F_{min}$ can also be estimated by the maximal distance range (ΔR) decided by the possible water depths of the measuring spot during waterlogging disasters:

(9) $$\Delta F_{d}=\frac{BW\times 2\Delta R}{T_{chirp}\times c\times f_{s}\;}.$$

Another significant parameter to be initialized is the step size of digital frequency-sweep $F_{step}$, which determines the measuring precision $R_{des}$ by the following equation:

(10) $$R_{des}=\frac{F_{step}\times f_{s\;}\times T_{chirp}\times c}{2\times BW}.$$

Decreasing step size realizes more precise distance measuring but causes an increase in time complexity. Notice that $F_{step}$ can not be too small since it is meaningless when the measurement accuracy is obviously smaller than the error. Therefore, an optimal step size can balance the accuracy of distance measurement and time complexity of the algorithm.

#### 2.2.2. Phase Range

When using cross correlation between the single-tone signal and the sampled IF signal, the information of phase of the sampled IF signal is useless for distance calculation in this algorithm. In Equations (5) or (6), the information of phase means the value of x when the results of cross correlation $R_{fh}\left(x\right)$ reaches the maximum. What is needed in this algorithm is the crest value of $R_{fh}\left(x\right)$. According to Equation (3), the initial phase of the IF signal ($\varphi_{0}$) is the difference between the phase of the TX chirp and the phase of the RX chirp at the time instant corresponding to the start of the IF signal:

(11) $$\varphi_{0}=2\pi f_{c}\frac{2R}{c}.$$

when the distance range is estimated, the phase $\varphi_{d}$ of the sampled IF signal can be calculated by the following equation:

(12) $$\varphi_{d}=\frac{f_{c}\times T_{chirp}}{BW}\times f_{s}.$$

By determining the phase when using cross correlation, the time complexity is reduced from $O\left(n^{2}\right)$ to O(n).

### 2.3. Modification of the SFCC Algorithm

To reduce computation complexity for the SFCC algorithm, we modified the fitting method of single-tone signal of different frequency in the algorithm.

When SFCC is transplanted to the control center, the single-tone signal is realized by piecewise linear fitting, thus achieving fast calculation speed and taking small storage space [31]. Only 30 data points need to be stored to make precision less than $1\times 10^{-3}$, while 93 need to be stored to make precision less than $1\times 10^{-4}$, which is very suitable for running on the control board with limited computing power and storage space.

The above analysis demonstrates that the noise and other interference signals at each frequency point correlative to the non-reflection point may adversely affect the results of cross correlation. To reduce interference, the IF signal can be filtered before cross correlation by a digital band-pass filter generated by MATLAB according to the frequency range determined by the application scene of the radar. This process can be left out if the interference is acceptable.

### 2.4. General Description

**Figure 3:**
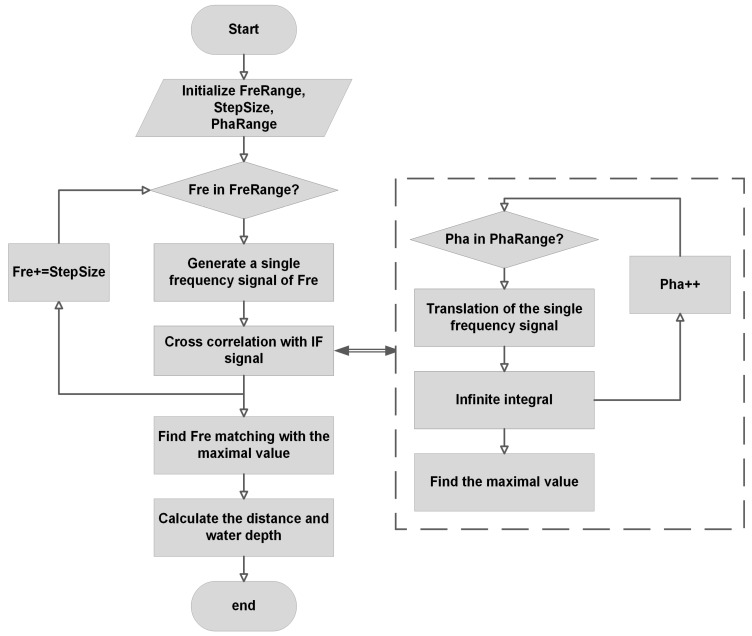
SFCC flow chart.

The flowchart is shown in Figure 3 to explain the SFCC algorithm. The initialization of the SFCC algorithm is operated first as described in Section 2.2 according to the specific application scenario. Then, generate single-tone signals successively and calculate with the sampled IF signal using cross correlation, during which the maximal value is found in the determined phase range. Finally, compare the maximum values of the cross-correlation calculation results of different single-tone signals and the sampled signal, and then take out the value of the frequency of the single-tone signal corresponding to the maximum value as $F_{d}$ to calculate the accurate distance from the surface of water to radar ($R_{s}$) by the following equation:

(13) $$R_{s}=\frac{F_{d}\times f_{s\;}\times T_{chirp}\times c}{2\times BW}.$$

Finally, calculate water depth when subtracted from the value of distance to the ground.

## 3. Event-Driven Scheme

In order to sustain the long work time for the waterlogging sensor, the power of the sensor should be as low as possible. Since the radar is only turned on during the sampling period, the power consumption is directly related to the measuring frequency. However, reducing the measurement frequency can affect the detection of rapid water depth change, especially when the rain is heavy. In order to not only achieve the low power consumption of the system, but also ensure that the system can reflect the change of water depth in time when the water level rises rapidly or may rise rapidly, we design an algorithm to control sampling frequency based on ambient temperature and humidity.

**Figure 4:**
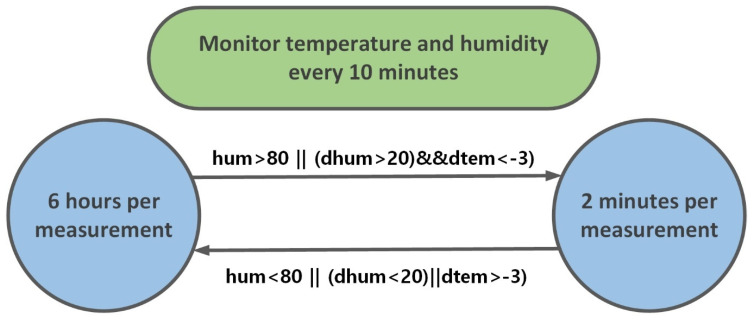
Block diagram of the event-driven algorithm.

The idea of the algorithm is to determine the frequency to run the radar and the control board once every 6 h in a normal situation and maintain the low power consumption mode for the rest of the time. Meanwhile, measure and obtain values of current temperature and humidity every 10 min, and calculate the changes compared with standard values of the same period in the locality, which can be obtained according to the previously measured data over the past few days. If the current humidity is higher than 80%, increase the sampling frequency to collect water depth data every 2 min. If the current humidity is more than 20% higher than the standard value and the temperature is more than 3 °C lower than the standard value, the sampling frequency will also be increased to collect water depth data every 2 min to ensure that the system can monitor the change of water level in time. The algorithm block diagram is shown in Figure 4.

The algorithm receives the current temperature (tem) and the current humidity (hum) as input and calculates the changes in temperature (dtem) and humidity (dhum) compared with standard values of the same period in the locality according to the previously saved data.

In the system, described in detail in the next section, the radar module needs 1.03 A power-supply current in the state of collecting data, and the power consumption is about 5.15 W, while the working current is about 0.77 A and the power consumption is about 3.85 W when the control center runs the SFCC algorithm. When the wireless communication module’s on-chip processor works normally and the Internet of things communication module performs wireless data exchange with the cloud platform, the working current is about 40 mA and the power consumption is about 0.2 W. However, in the standby mode the power supply current required by the wireless communication module is only within 10 μA and the current required by the radar module is only 500 μA. Therefore, by applying this event-driven algorithm, the power consumption in usual circumstances can be lowered to less than 1% of the continuous working mode.

## 4. Measurement System

**Figure 5:**
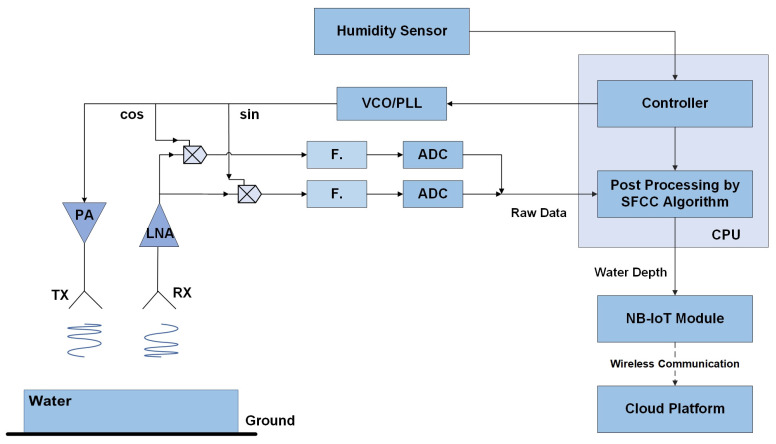
A block diagram of the system.

To prove the practicability of our approach to measuring water depths, a complete system is built according to the block diagram shown in Figure 5.

### 4.1. FMCW Radar Configuration

The FMCW radar module includes a 76–81 GHz millimeter wave development board, which integrates transmit (TX) and receive (RX) radio frequency (RF) components, clocks and other analog components, as well as analog-to-digital converters (ADC), microcontroller (MCU) and other digital components, and a real-time data-capture board connected to the above-mentioned board to capture its raw data. Table 1 shows the configuration parameters in detail.

### 4.2. System Description

#### 4.2.1. Overview

**Figure 6:**
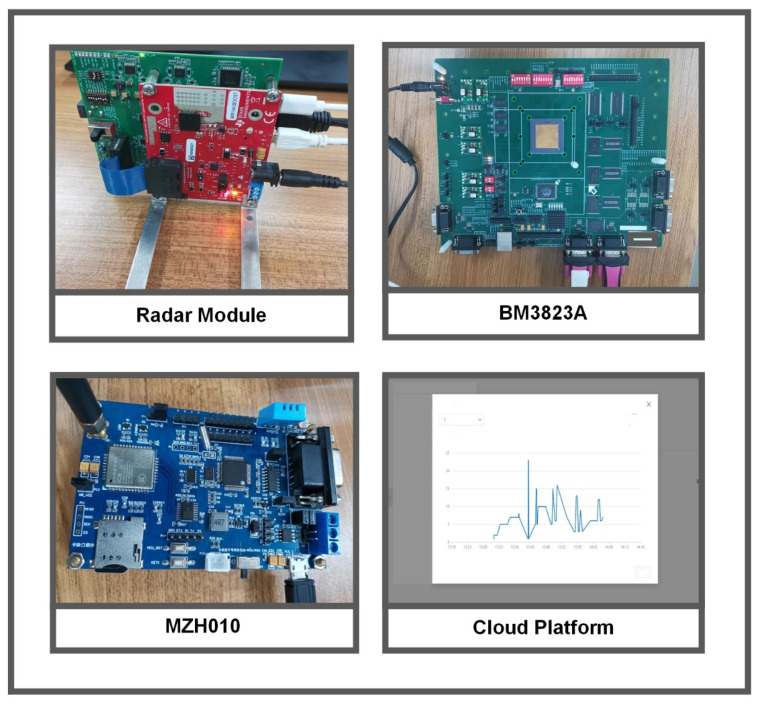
Measurement modules of the system.

The measurement system consists of the millimeter wave transmitting and receiving module IWR1443BOOST [32], a data acquisition module DCA1000EVM [33], a processor module BM3823A and a wireless communication module MZH010 NB-IoT. Figure 6 depicts the system-level diagram, showing photographs of the four modules in the system.

**Figure 7:**
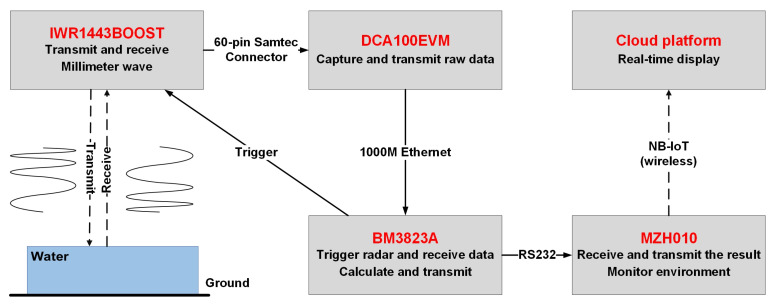
Overall architecture of the measuring system.

The overall architecture of the system is shown in Figure 7 below:

The IWR1443BOOST is connected to DCA1000EVM through a 60-pin high-speed connector. The latter transmits the data to BM3823A through the network cable. Then, BM3823A uses the DuPont line to connect its GPIO port to the corresponding radar interface to generate the trigger level to start the acquisition of data. After a while, the value of the distance calculated by the SFCC algorithm is sent to the MZH010 NB-IoT through the RS232 serial port line. BC26 of the development board then transmits the value to the cloud platform, so as to realize real-time monitoring of urban waterlogging depth. In order to reduce power consumption, the narrowband IOT module collects the temperature and humidity data of the current environment, intelligently judges the timing of measuring water depth, and controls the processor to trigger radar and calculate the water depth.

According to the architecture diagram, in addition to the power lines of each module, only one gigabit network cable, one serial port cable, two DuPont lines for transmitting trigger level and one row of 60-pin Samtec connecting lines are required to work normally. No wired connection is required between the whole system and the PC.

#### 4.2.2. Data Transmission Based on UDP

The process of data acquisition from FMCW millimeter-wave radar is very fast, with the transmission rate of ADC being more than 700 Mbps. Therefore, category five and above network cables and Gigabit Ethernet can be used to receive data.

**Figure 8:**
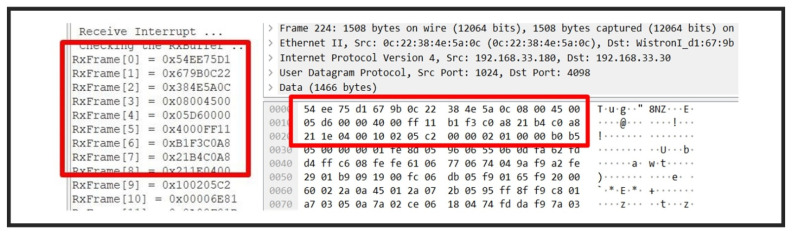
Verification of the data received by the control board.

Data transmission is based on User Datagram Protocol (UDP). Figure 8 shows the consistency between the data received by the control board and the data captured by the PC.

**Figure 9:**
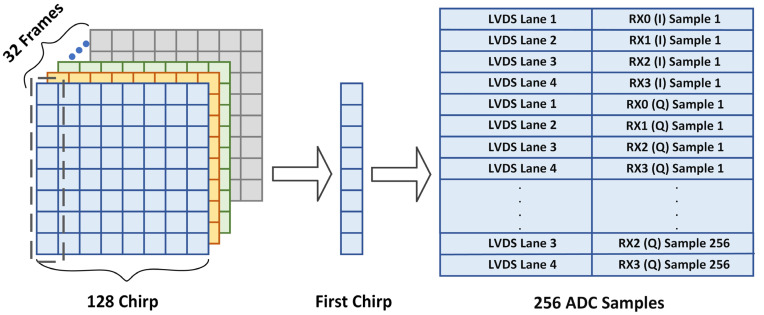
Details of data arrangement.

The UDP datagram transmitted from the radar needs to be reorganized. The total size of the data of the sampled signal is 256 × 16 × 2 × 4 = 32,768 bits, since there are 256 sampling points with each sampling point being a 16-bit number, while four receiving antennas are enabled to make double channel acquisition. Therefore, UDP datagrams need to be combined in the same order by the control board. The specific data arrangement is shown in Figure 9.

### 4.3. System Verification

**Figure 10:**
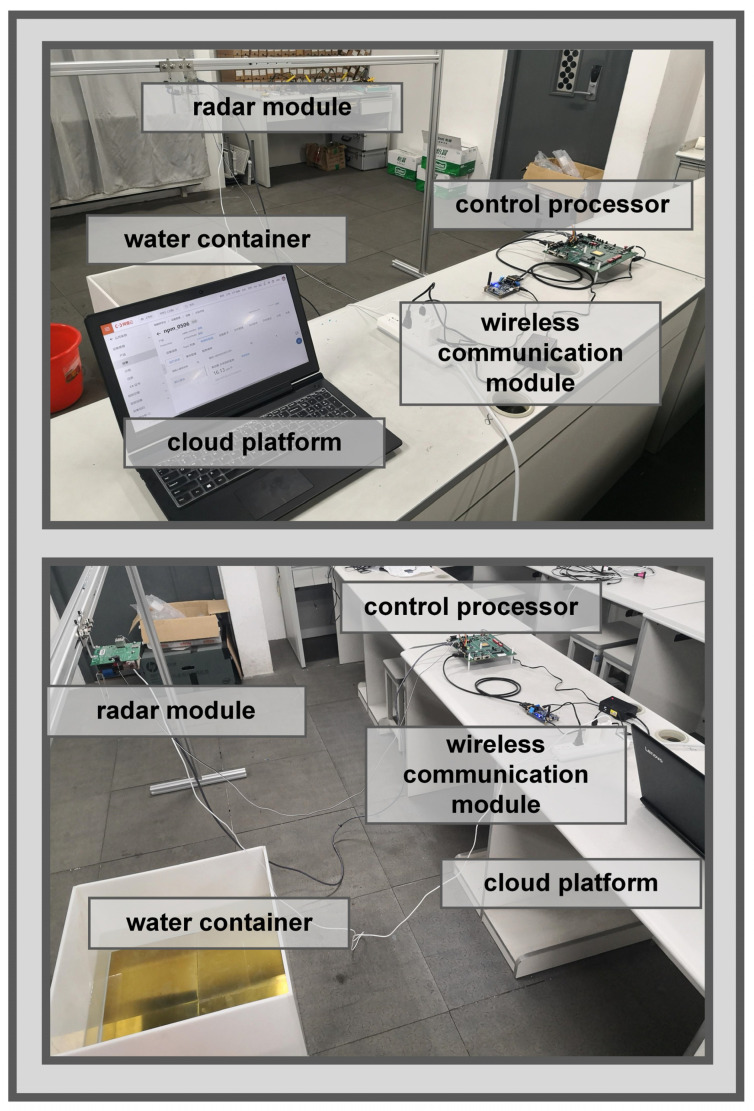
Overall construction of the system photographed from two different angles.

The overall construction of the system is shown in Figure 10. According to the system architecture diagram and module introduction, a practical system is built after the development boards are configured and connected with each other through interfaces.

The wireless communication module in the system can transmit the measurement results to the cloud platform in real time, which is convenient for data monitoring and post-processing. The change of the depth of water in the actual application scenario is simulated and the sensitivity of the system to this change and the measurement accuracy is tested. The result of our experiment in Table 2 shows that the system can capture small changes in water depth, and the error between the measurement results and the actual values of the water depth when the water surface is stable is kept within 5 mm, with good overall performance.

## 5. Conclusions

To satisfy the requirement of real-time estimation of urban waterlogging depths, a novel and practical system designed to be feasibly installed within cities and equipped with a humidity sensor, a processor module and a display module is proposed in this paper. It is based on mmWave FMCW Radar to perform robust and accurate measurement. The algorithm that runs on the processor is a new algorithm named SFCC designed to improve distance resolution and lower time complexity in accordance with the edge computing concept, suitable for limited computing power and storage space, with time complexity being O(n) and an estimated depth error of less than 5 mm.

The system has the following functional advantages:

- Wide range—frequency-modulated continuous-wave millimeter wave radar, which can detect aims far away;
- Low error—original distance calculation algorithm SFCC with measurement error of less than 5 mm;
- Real-time monitoring and display on a cloud platform based on narrowband Internet of things technology;
- Bandwidth saving—in accordance with edge computing and low bandwidth requirements, convenient for multi-channel networking.

Meanwhile, there are also product advantages as follows:

- Stability—the radar and anti-radiation processor ensuring stable operation in various conditions;
- Low power consumption—event-driven monitoring actualizing high efficiency and energy saving;
- Small volume—possible for the volume of the core module to be reduced to within 10 cm × 10 cm × 5 cm;
- Low cost—after further optimization, possible for the installation cost to be within 100 dollars.

## Figures and Tables

**Table 1:** Configuration parameters of the radar module.

S. No.	Configuration Parameter	Value
1	Starting Frequency	77 GHz
2	BW	3.8991 GHz
3	Slope	64.985 MHz/μs
4	Number of RX	4
5	Number of TX	3
6	Number of ADC samples	256
7	Number of chirp loops	128
8	Number of the frame	32
9	ADC sampling rate	5120 MHz

**Table 2:** Measuring results and mean errors of water depths.

No.	Measuring Result (cm)	Ture Depth (cm)	Mean Error (mm)
1	16.6058	16.6	0.058
2	14.6032	14.4	2.032
3	12.365	12.5	1.35
4	3.53	4	4.7
5	2.2342	2.2	0.342
6	0.3494	0	3.494
